# Myocarditis and pericarditis in association with COVID-19 mRNA-vaccination: cases from a regional pharmacovigilance centre

**DOI:** 10.21542/gcsp.2021.18

**Published:** 2021-10-30

**Authors:** Ioanna Istampoulouoglou, Georgios Dimitriou, Selina Späni, Andreas Christ, Barbara Zimmermanns, Sarah Koechlin, Oliver Stoeckmann, Clemens Winterhalder, David Marono, Valeriu Toma, Anne B Leuppi-Taegtmeyer

**Affiliations:** 1 Department of Clinical Pharmacology & Toxicology, University Hospital and University of Basel, Switzerland; 2 Emergency department, Cantonal Hospital Aarau, Switzerland; 3 Hospital Pharmacy, Cantonal Hospital Basel Landschaft, Liestal, Switzerland; 4 Intensive Care Unit, Cantonal Hospital Basel Landschaft, Liestal, Switzerland; 5 Regional Pharmacovigilance Centre Basel, University Hospital Basel, Basel, Switzerland; 6 Department of Internal Medicine, University Hospital Basel, Basel, Switzerland; 7 Medical Outpatient Department, University Hospital Basel, Basel, Switzerland; 8 Swissmedic, Swiss Agency for Therapeutic Products, Bern, Switzerland

## Abstract

In this article we summarize suspected adverse events following immunization (AEFI) of pericarditis, myocarditis and perimyocarditis that were reported by our regional pharmacovigilance centre after COVID-19 mRNA-vaccination and discuss their association with these vaccines. Seventeen cases were reported between March and July 2021. Of these, nine had perimyocarditis, five myocarditis and three pericarditis. Twelve patients were male (71%). The median age was 38 years (range 17–88). The most commonly observed presenting symptom was acute chest pain (65%). While 47% of the patients were previously healthy, 53% had at least one pre-existing comorbidity, with hypertension being the most prevalent (24%). The European Society of Cardiology diagnostic criteria for the reported AEFIs were fulfilled in twelve cases (71%). The AEFIs occurred after the first vaccine dose in six cases (35%), after the second vaccine dose in ten cases (59%) and after both doses in one case (6%). The median latency of all AEFIs taken together was 14 days (range 1–28) after the first vaccination and 3 days (range 1–17) after the second one. All patients except one were hospitalized (94%) with a median length of stay of 7.5 days (range 3–13). The majority of patients (*n* = 11, 65%) did not experience any complications, and 13 (77%) of the patients had recovered or were recovering at the time of discharge. In 16 of the 17 cases (94%), the association between the AEFI and mRNA-vaccination was considered possible by the pharmacovigilance centre.

## Introduction

The prerequisite for cellular entry of the SARS-CoV-2 virus is the binding of the virus to the membrane-bound form of the angiotensin converting enzyme 2 (ACE2), which enables the complex to be internalized by the host cell^1^. ACE2 is expressed by epithelial and endothelial cells of the respiratory tract, as well as by several extrapulmonary tissues including heart, kidney, brain, and gut^1–3^.

Therefore, the respiratory and other organ systems, including the cardiovascular system, can be both directly (by the SARS-CoV-2 virus itself), and indirectly (as a result of a virus-induced systemic inflammatory cytokine storm with endothelial dysfunction) affected^2,4^. Like in other viral illnesses, inflammatory diseases of the pericardium (pericarditis) or myocardium (myocarditis) or both (perimyocarditis) can occur^4–7^.

According to current knowledge about cardiac AEFIs after vaccination with non-mRNA-vaccines, (peri-)myocarditis is a recognized adverse drug reaction (ADR) of smallpox, influenza and hepatitis B vaccines^8,9^. Post-vaccination pericarditis without myocardial involvement has also been described in the literature, but appears to be very rare^10–12^.

Recently, significantly more cases of pericarditis and myocarditis than expected have been observed after COVID-19 vaccination with mRNA-vaccines during post-marketing pharmacovigilance surveillance. Therefore, the US Food and Drug Administration (FDA) followed by the European Medicines Agency (EMA), and then Swissmedic in Switzerland, have included myocarditis and pericarditis as very rare ADRs in the product information for the mRNA-vaccines marketed by Moderna and Pfizer-BioNTech^13,14^.

In this article we summarize the cases of pericarditis, myocarditis and perimyocarditis that were observed in seventeen patients after vaccination with an COVID-19 mRNA-vaccine and discuss their association with the vaccination. The cases were reported to our regional pharmacovigilance centre between March and July 2021. There, pharmacovigilance assessments were performed according to the World Health Organization (WHO) criteria^15^.

A spontaneous pharmacovigilance report is a snapshot that provides information on whether a drug or vaccine could in principle be associated with a suspected adverse effect. It also assesses the degree of association between the drug and the suspected ADR, namely as being certain, likely, possible, unlikely, or unclassifiable. It must be noted that a spontaneous report cannot answer the question whether the symptoms observed in a particular patient are wholly attributable to the drug intake. This question can only be answered by an individual medical opinion. Nevertheless, in Switzerland medical professionals and all those who manufacture, administer or dispense therapeutic medicinal products—including medical personnel who are authorized to do so—are obliged by law to report the occurrence of ADRs to the national regulatory authority for medicinal products, Swissmedic.

## Methods

We examined the internal database of the Regional Pharmacovigilance Centre (RPVC), Basel for anonymous pharmacovigilance reports regarding pericarditis, myocarditis and perimyocarditis in connection with a COVID-19 mRNA-vaccination and extracted demographic and clinical data from the completed reports. The RPVC in Basel is one of six RPVCs in Switzerland (current population 8.5 million), and covers the northwestern region, which is largely German-speaking. We also determined whether diagnostic criteria for these inflammatory cardiac conditions were fulfilled by examining the concomitantly reported information. We used the European Society of Cardiology guidelines for this assessment (Boxes 1 and 2)^18,19^. We counted the number of cases of suspected drug-induced myocarditis, perimyocarditis and pericarditis, which were reported to the RPVC during the last ten years and recorded the suspected causative agents.

**BOX 1:** Diagnostic ESC-criteria for clinically suspected myocarditis (adapted from^19^) 
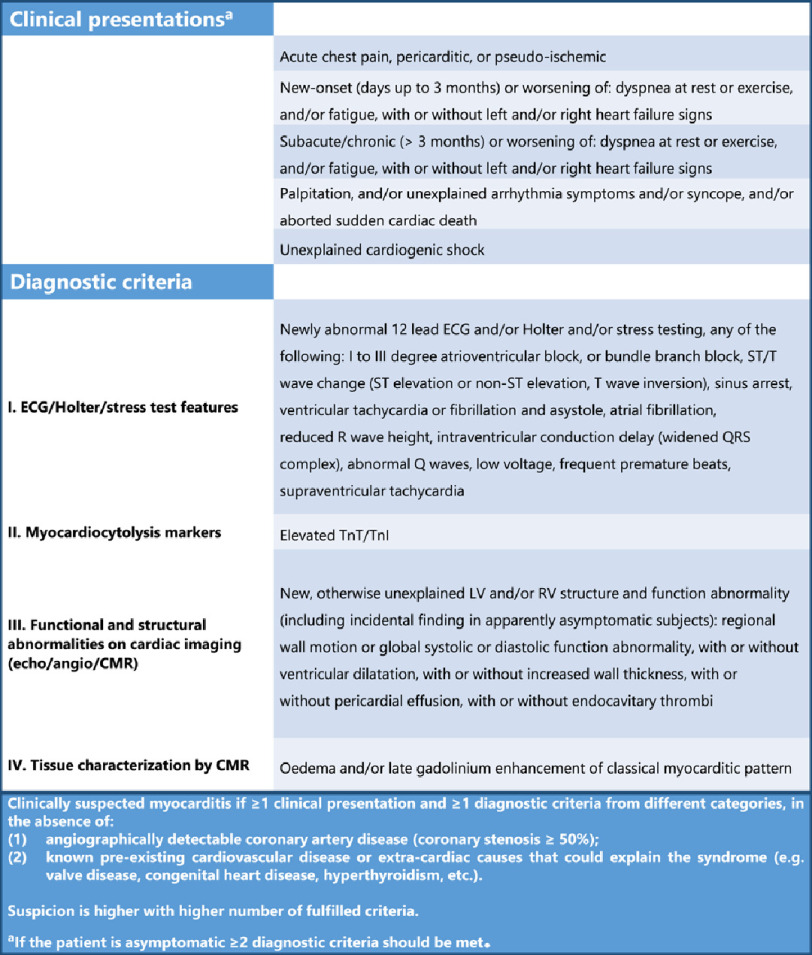


**Table 1:** Demographic, clinical, and laboratory characteristics of the clinical cases and adverse events following immunization (AEFI) details. Troponin T hs normal range <14 ng/L, Troponin I hs normal range <19 ng/L.

**Patient number**	**Age, Sex**	**Symptoms**	**Comorbidities**	**COVID-19 mRNA-vaccine**	**Cardiac Diagnostics**	**Reported AEFI**	**Diagnostic criteria fulfilled^18,19^**	**Latency to AEFI**	**Management**	**Complications (arrhythmia, heart failure)**	**LOS (days)**	**AEFI outcome at time of PV report**
1.	20, male	Acute chest pain	None	Moderna	TnI hs: abnormal (max. 13654.5 ng/L) ECG: abnormal TTE: normal	Myocarditis	Yes - partially	3d after 2nd dose	ACE-inhibitor	None	6	Recovering
2.	38, male	Palpitation, dizziness	Atopic dermatitis	Pfizer-BioNTech	TnT hs: abnormal (40 ng/L) ECG: abnormal TTE: abnormal (LVEF 45%) CMR: abnormal	Myocarditis	Yes –all	25d after 1st dose	ACE-inhibitor, β-blocker, SGLT2 inhibitor, aldosterone antagonist	Non sustained VT, SVT and heart failure	10	Recovering
3.	23, female	Acute chest pain	Previous erythema migrans, juvenile arthritis (in childhood)	Moderna	TTE: abnormal (LVEF 56%) TnT hs: normal ECG: normal bedside sonography: normal	Suspected myocarditis/ musculoskeletal chest pain	Yes - partially	17d after 1st dose	ibuprofen	None	3	Recovering
4.	44, female	Fatigue, acute chest pain, fever	New simultaneous diagnosis of Lyme disease with acute Lyme carditis	Moderna	TnT hs: abnormal (384 ng/L) CMR: abnormal ECG: normal Coronary angiography: normal TTE: not conclusive (LVEF 54%)	Myocarditis	Yes - all	Ca. 28d after 1st dose	Antibiotic therapy	None	7	Recovering
5.	34, male	Acute chest pain	None	Moderna	TnT hs: abnormal (568 ng/L) ECG: abnormal CMR: abnormal TTE: normal (LVEF 60%)	Myocarditis	Yes –all	4d after 2nd dose	ACE-inhibitor, β-blocker	None	5	Recovering
6.	86, male	Fatigue	CAD, hypertension	Moderna	TnT hs: abnormal (74 ng/L) ECG: abnormal TTE: abnormal (LVEF 50–55%)	Perimyocarditis	Yes - partially	1d after 2nd dose	unknown	None	8	Recovering
7.	23, male	Palpitation, acute epigastric pain radiating to the throat, fever (38.7 ° C)	Renal transplant, gout, hypertension	Pfizer-BioNTech	TnT hs: abnormal (max. 2010 ng/L) ECG: abnormal CMR: abnormal TTE: normal	Perimyocarditis with pericardial effusion	Yes –all	10d after 1st dose	antibiotic therapy	None	10	Recovered without sequelae
8.	70, male	Dyspnea, acute chest pain	Multimorbid*, new diagnosis of CAD 1d after ADR	Moderna	TnT hs: abnormal (max. 1139 ng/L) ECG: abnormal Coronary angiography: abnormal TTE: normal (LVEF 60%) CMR: normal	Perimyocarditis with pericardial effusion	Yes - partially	<24 h after the 1st dose	ibuprofen, colchicine	None	13	Recovering
9.	88, female	Palpitations, dyspnea on exertion, fever (38.5 ° C)	Hypertension, obesity	Pfizer-BioNTech	(CT-scan: abnormal)	Perimyocarditis with pericardial effusion	Yes - partially	17d after the 2nd dose	ibuprofen, colchicine, antibiotic therapy, pericardiocentesis with pericardial catheter	Right heart failure	9	Recovered without sequelae
10.	57, male	Acute chest pain	None	Moderna	TnT hs: abnormal (52 ng/L) ECG: abnormal CMR: abnormal TTE: normal (LVEF 55%)	Perimyocarditis with pericardial effusion	Yes –all	2d after 2nd dose	ACE-inhibitor	None	5	Recovering
11.	19, male	Acute chest pain	None	Moderna	TnT hs: abnormal (max. 788 ng/L) ECG: abnormal TTE: abnormal (LVEF 52%) CMR: abnormal	Perimyocarditis with pericardial effusion	Yes –all	3d after 2nd dose	ACE-inhibitor, β-blocker, ibuprofen	VE, intermittent bigeminus	10	Recovered without sequelae
12.	69, female	No symptoms attributable to perimyocarditis	Suspected simultaneous manifestation of systemic rheumatic disease such as polymyositis	Moderna	TnT hs: abnormal (347 ng/L) CMR: abnormal ECG: normal TTE: normal (LVEF 60%)	Perimyocarditis with pericardial effusion	Yes - all	1d after 2nd dose	Loop diuretic, aldosterone antagonist, β-blocker	None	10	Recovering
13.	17, male	Acute chest pain	None	Pfizer-BioNTech	TnT hs: abnormal (max. 3477 ng/L) ECG: abnormal TTE: abnormal (LVEF 50%) CMR: abnormal	Perimyocarditis with pericardial effusion	Yes –all	1d after 2nd dose	ACE-inhibitor, β-blocker	Left Heart failure	5	Recovering
14.	19, male	Acute chest pain	None	Moderna	TnT hs: abnormal (643 ng/L) ECG: abnormal CMR: abnormal TTE: normal (LVEF 61%)	Perimyocarditis with pericardial effusion	Yes –all	3d after 2nd dose	ACE-inhibitor, β-blocker	None	3	Recovering
15.	33, female	Acute chest pain, palpitations	Previous hepatitis B infection, hepatic cyst, normochromic normocytic anemia, recurrent urticaria of unclear etiology	Pfizer-BioNTech	ECG: abnormal TnT hs: normal	Suspected subacute pericarditis	Yes –all	1d after 1st dose	Ibuprofen, colchicine	None	0	Recovering
16.	61, male	Unknown	recurrent idiopathic pericarditis (diagnosis after 4th episode), major depressive disorder, hypertension, asthma-COPD-overlap with chronic bronchitis, obstructive sleep apnea, obesity, methadone substitution	Unknown	Unknown	Pericarditis	Unknown	14d after 1st dose	Ibuprofen, colchicine	None	8	Recovering
					Unknown	Pericarditis	Unknown	10d after 2nd dose	Ibuprofen, colchicine and percutaneous pericardiocentesis	None	11	Recovering
					17.	71, male	Acute chest pain, fatigue, fever (38.9 ° C), new onset dyspnea	Arrhythmia (not further defined)	Moderna	TTE: abnormal (LVEF unknown) ECG: unknown	Pericarditis with exudative pleuritis	Yes - all	14d after 2nd dose	Ibuprofen, colchicine, β-blocker, rivaroxaban, amiodarone, PPI	unknown	7	Partially recovered

**Notes.**

11*: ischaemic and rhythmogenic cardiomyopathy, paroxysmal atrial fibrillation, type 2 diabetes mellitus, obesity, hypercholesterolemia, myelofibrosis, previous allogeneic hematopoieticl stem cell transplantation, chronic renal failure (KDIGO stage G2) sensorimotor axonal demyelinating polyneuropathy, obstructive sleep apnea, major depressive disorder.

ACE angiotensin-converting enzyme ADR adverse drug reaction CAD coronary artery disease CT scan computed tomography scan ECG electrocardiography LOS length of hospital stay CMR cardiovascular magnetic resonance LVEF left ventricular ejection fraction PPI proton pump inhibitor PV pharmacovigilance TnT hs Troponin T high sensitivity TnI hs Troponin I high sensitivity TTE transthoracic echocardiogram VE ventricular extrasystoles VT ventricular tachycardia

CMR: cardiovascular magnetic resonance; CT: computed tomography; ECG: electrocardiogram; LV: left ventricular; RV: right ventricular; TnT/I: Troponin T/I

**BOX 2:** Diagnostic ESC-criteria for pericarditis (adapted from^18^)


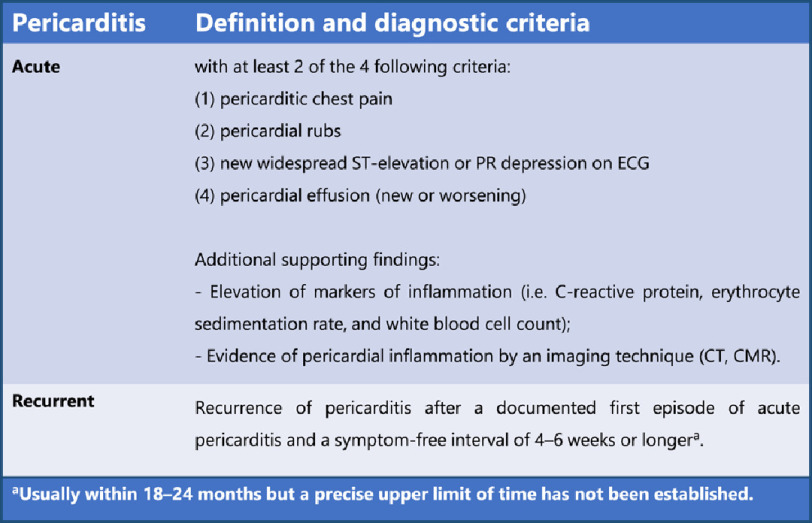


CMR: cardiovascular magnetic resonance; CT: computed tomography; ECG: electrocardiogram.

We also examined data from VigiBase, the WHO global database of individual case safety reports. A detailed description of VigiBase can be found in the publication by Lindquist^16^. We searched VigiBase using VigiAcess for the active ingredient “COVID-19 vaccine”^17^. This term includes all vaccinations against SARS-CoV-2 virus that are available worldwide.

## Results

The relevant demographic, clinical, and laboratory information for each patient case and details about each adverse event following immunization (AEFI), as well as the outcome and ultimate pharmacovigilance assessment, are given in Table 1. The diagnosis of myocarditis or pericarditis was made according to the diagnostic criteria of the European society of cardiology (ESC), which are presented in Boxes 1 and 2^18,19^.

### Myocarditis cases

The median age of the five patients with myocarditis was 34 years (range 20–44) and three of them were male (60%), also with a median age of 34 years (range 20–38). The most frequent symptom was acute chest pain, which was present in 80% of the patients. Three patients (60%) had no pre-exiting comorbidities, but one of them was simultaneously diagnosed with Lyme disease with acute Lyme carditis (case 4). Four patients (80%) were vaccinated with the mRNA-vaccine marketed by Moderna and one (20%) with Comirnaty^®^.

The ECG was abnormal in three cases (60%). Troponin levels were abnormal (mean TnT 330.7 ng/L ± 218.8) in four of five patients with myocarditis (80%) and in one patient (case 3) TnT hs was normal.

Cardiac MRI was performed in three of five patients as appropriate imaging for the myocarditis diagnosis (60%), and was suggestive of this diagnosis (100%) in all three cases. Four of the five AEFIs (80%) were correctly reported as partially or completely meeting the diagnostic criteria of myocarditis. Among the five myocarditis cases, three occurred after the first mRNA-vaccination with a median latency of 25 days (range 17–28) and two after the second with a median latency time of 3.5 days (range 3–4). The most commonly used treatment was a cardioprotective therapy with an ACE-inhibitor (60%), followed by a β-blocker (40%). The median length of stay was 6 days (range 3–10 days) and no complications were seen in 80% of cases. One patient experienced non-sustained ventricular tachycardia, supraventricular tachycardia and heart failure. All patients were discharged in a “recovering” state. All cases were evaluated as being possibly related to mRNA-vaccination by the pharmacovigilance centre.

### Perimyocarditis cases

The median age of the nine patients with perimyocarditis was 57 years (range 17–88) and seven of them (78%) were men with a median age of 23 years (range 17–86). Acute chest pain was the most common presenting symptom (56%), followed by palpitations, fever and dyspnea (each in 20%). One patient had acute epigastric pain (case 7) and another had no symptoms attributable to perimyocardits (case 12). Five of the patients (56%) had no pre-existing comorbidities. In patients with pre-existing conditions (44%), hypertension was the most prevalent comorbidity (75%). One patient was diagnosed with coronary artery disease one day after the reported AEFI (case 8). Furthermore, the treating physicians in case 12 suspected a concurrent manifestation of a systemic rheumatic disease such as polymyositis.

Six patients (67%) were vaccinated with the mRNA-vaccine marketed by Moderna and three (33%) with Comirnaty^®^. ECG findings were known in eight of nine patients with perimyocarditis; of which seven cases (88%) had an abnormal ECG. Troponin levels were known and abnormal (mean 1066.25 ng/L ± 1089.4) in eight of nine patients with perimyocarditis (89%). Cardiac MRI was performed in seven of nine patients with perimyocarditis as appropriate imaging (78%) and in six cases it was suggestive of this diagnosis (86%). Six of the nine AEFIs (67%) were correctly reported as they completely or partially fulfilled the diagnostic criteria of perimyocarditis.

In one patient (case 9) the only diagnostic test performed was a CT scan, which is not part of the standard imaging for perimyocarditis. Of all clinical cases of perimyocarditis, only two (22%) occurred after the first mRNA-vaccination with a median latency of 0 (<24 h) and 10 days, respectively. The majority of cases (78%) occurred after the second dose with a median latency time of 2 days (range 1–17). The most commonly used treatment was a cardioprotective therapy with an ACE-inhibitor or a β-blocker (each 44%), followed by a combination of these two drugs or ibuprofen as anti-inflammatory therapy (each 33%). The median length of stay was 6 days (range 3–13) and three patients (20%) experienced complications (one case each of right heart failure, ventricular extrasystoles and left heart failure, respectively). Six patients were discharged in a “recovering” health state (67%) and three recovered fully during hospitalization (33%). All clinical cases with perimyocarditis were evaluated as possibly related to mRNA-vaccination by the pharmacovigilance centre.

### Pericarditis cases

The total median age of the three patients with pericarditis was 61 years (range 33–71) and two of them (67%) were men. Two patients (67%) presented to the emergency department with acute chest pain and various accompanying symptoms. The symptoms of the third patient (case 16) remained unknown. All patients had at least one comorbidity. One patient was vaccinated with the mRNA-vaccine marketed by Pfizer-BioNTech (tozinameran - Comirnaty^®^) and one with the vaccine marketed by Moderna (now called elasomeran, Spikevax^®^). In case 16 the name of the administered mRNA-vaccine as well as the performed cardiac diagnostic tests were unknown.

The electrocardiogram (ECG) was abnormal in two cases and high sensitivity troponin T (TnT hs) level was measured as not elevated in one case. While two of the three AEFIs (67%) fulfilled the diagnostic criteria of pericarditis, the third case was unclear as the report was completed by the patient himself. Case 16 had known recurrent pericarditis, which was diagnosed after the fourth episode, with two episodes occurring a few days after the first and second mRNA-vaccination, respectively.

Of the total of four pericarditis episodes in these three patients, 50% took place after the first mRNA-vaccination with median latency of 7.5 days (range 1–14) and 50% after the second, with a median latency time of 12 days (range 10–14). For the management of pericarditis, all three patients received anti-inflammatory therapy with ibuprofen and colchicine. The median length of hospital stay was 7.5 days (range 0–11), with no complications and a “recovering” health outcome reported in two cases. In one patient (case 17), it was unknown whether there were complications, but his health condition was reported as “partially recovered” at the time of reporting. Two of the three clinical cases with pericarditis were evaluated as being possibly related to mRNA-vaccination (67%); case 16 was considered unlikely by the pharmacovigilance centre.

**Figure 1.**
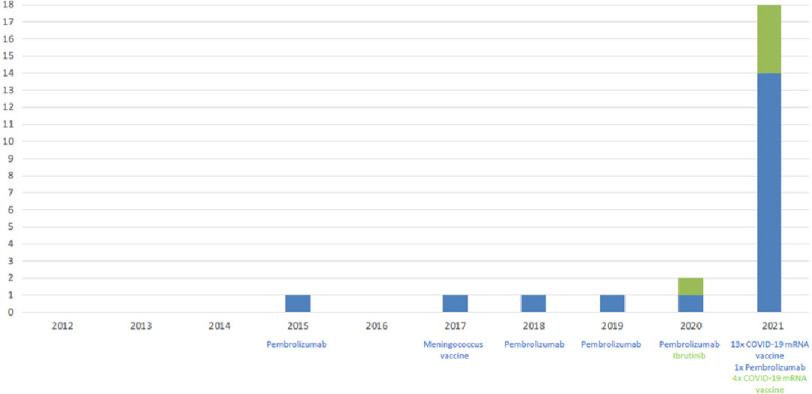
Number of cases of suspected drug- or vaccine-induced myocarditis, perimyocarditis and pericarditis reported to the RPVC from 2012 until July 2021. Blue bars and blue lettering indicate the number of myocarditis and perimyocarditis cases, green bars and green lettering indicated the number of pericarditis cases.

The number of myocarditis, perimyocarditis and pericarditis cases reported to the RPVC during the past ten years is shown in Figure 1.

## Discussion

### Current study findings

Cases of pericarditis, myocarditis and perimyocarditis after mRNA-vaccination were first sent to our regional pharmacovigilance centre in March 2021, two months after the mRNA-vaccines were licensed in Switzerland. We summarize the first seventeen reported cases in this article. Although pharmacovigilance data do not give any information about incidence, they are essential for signal-detection.

At our RPVC we observed a dramatic increase in cases of suspected drug- or vaccine induced myocarditis, perimyocarditis and pericarditis in 2021 compared to the proceeding nine years (Figure 1). This increased incidence of reported cases was associated with COVID-19 mRNA vaccination.

Pharmacovigilance reports of AEFIs must always be assessed in the context of the exposure group –number of exposed people, age and co-morbidities. Between January and July 2021, approximately 9 million doses of mRNA-vaccines were administered in Switzerland (approximately 3 million Comirnaty^®^ and 6 million Covid-19 vaccine Moderna^®^), with older and other high-risk patients being vaccinated first^20^. This is a likely reason, why the very rare AEFI of (peri-)myocarditis - which seems to predominantly affect younger people - was not observed straight away after licensing.

The Swiss product information for tozinameran (Comirnaty^®^, Pfizer-BioNTech) and elasomeran (Spikevax^®^, Moderna) were recently updated to include both pericarditis and myocarditis as very rare ADRs (<1 case per 10,000 vaccinated people), and a joint direct health professional communication (DHPC) relating to both tozinameran and elasomeran was issued^21^. This illustrates the role of post-marketing surveillance and spontaneous pharmacovigilance reports in improving vaccine safety for the whole population. According to the literature, these inflammatory heart diseases do not appear to be a typical adverse event following vaccination with the adenovirus vector-based vaccine marketed by AstraZeneca (Vakzevria^®^)^22^.

SARS-CoV-2 induced pericarditis and myocarditis are found predominantly in older men (62% and 58% respectively) with a median age of 50.4 and 51.6 years respectively^4,23^. In contrast, mRNA-vaccine induced myocarditis occurs mainly in otherwise healthy male adolescents and young adults aged 12–39 years^24–27^, whereas pericarditis is observed particularly in older male patients aged 46–69 years^22,28^. Our results regarding age and gender for all three AEFIs could confirm the existing literature data.

Underlying cardiovascular disease is a risk factor for developing cardiac injury in the setting of SARS-CoV-2 infection and is also associated with a higher mortality rate^2^. However—in contrast to pericarditis—it does not seem to play a major role in the development of mRNA-vaccine associated myocarditis^22,28^. In this regard, 60% of our myocarditis cases and 56% of perimyocarditis cases had no previous medical history, whereas all our patients with pericarditis had at least one comorbidity. In the literature, hypertension was found to be the most common (33%) concomitant disease in mRNA-vaccine induced myocarditis or pericarditis in patients with a medical history^4,22^. This observation was confirmed only in our perimyocarditis cases but not in our patient population with pericarditis, possibly due to the small number of these patients in our case series.

The typical and most frequently observed symptom of all these cardiac AEFIs is acute chest pain^24^, which was also the most commonly reported symptom in each of our three patient groups. In other studies, myocarditis was most often observed a few days after administration of the second mRNA-vaccination^22,24^ with a median latency time or time-to-onset of 2–3 days (range 1–5)^24^. This fact could only be observed in our patient population with perimyocarditis, whereas myocarditis cases occurred predominantly after the first vaccine dose and with a much longer mean latency. The most likely reason for this is the small sample size of just five cases of myocarditis. Taking both groups of patients with myocarditis and perimyocarditis together, the total median latency time was 3 days (range 1–17) after administration of the second dose, which is consistent with the existing literature. The symptoms of pericarditis appear to begin later with median time-to-onset of 20 days (range 6–41) after either the first or the second mRNA-vaccination^22,28^. Our observation of a median latency time of 7.5 days after the first and 12 days after the second dose is consistent with the above literature data.

According to a review of 61 clinical cases of myocarditis published in the medical literature, troponin levels were always elevated and cardiovascular MRI was always consistent with myocarditis; ECG changes (ST elevation, T wave changes) were not always present^24^. In most of our clinical cases of myocarditis and perimyocarditis, troponin levels were elevated (80% and 89% respectively), a cardiovascular MRI was suggestive of the diagnosis (100% and 86% respectively) and relevant ECG changes were present (60% and 88% respectively). Based on 39 published cases of pericarditis, troponin levels were always normal, whereas typical ECG changes (ST segment elevation, PR segment depression) were the leading diagnostic findings^22,28^. The troponin level was not known in two of our three pericarditis cases, making interpretation impossible. ECG findings were known and abnormal in two pericarditis cases, which is consistent with the existing literature.

The majority of patients with myocarditis, perimyocarditis or pericarditis require hospitalization. However, most patients recover after a few days with or without treatment, and are discharged without any, or only mild, symptoms^22,24,28^. The observations of the present study are consistent with this. Our total of seventeen patients survived and were discharged after a few days of hospitalization and treatment, and their health condition was reported as “recovering” or “recovered”.

### Pathomechanisms

Several possible mechanisms have been proposed for SARS-CoV-2 induced cardiac injury. Hypotheses include direct ACE2-mediated viral damage to cardiomyocytes and an indirect viral effect through endothelial inflammation (endothelialitis) as part of severe cytokine release syndrome with systemic inflammation^4,29^.

In the case of mRNA-vaccine induced myocarditis, it has been proposed that in people with a certain genetic predisposition, the otherwise less immunogenic modified spike protein coding mRNA molecule used in the vaccine, is detected as antigen. This leads to activation of an aberrant immune system response resulting in different immunological and inflammatory processes and consequently a systemic reaction to the vaccine^30,31^. However, the exact pathophysiology for developing cardiac inflammation in the context of a SARS-CoV-2 infection and the mechanism of an association with mRNA-vaccination are currently only poorly understood, or even still unknown^4,24^.

Currently available endomyocardial biopsy and heart autopsy findings in two patients with mRNA-vaccination induced myocarditis show a clearly inflammatory infiltrate with mixed pattern of T-cells and macrophages as well as eosinophils, B cells and plasma cells^32^. In comparison, the histological picture of SARS-CoV-2-induced myocarditis does not seem to be conclusive according to current literature, and the diagnosis can only be confirmed in 4.5% of highly selected endomyocardial biopsy or autopsy samples. In the majority of studied SARS-CoV-2-infected subjects, only nonspecific inflammatory infiltrates composed of increased interstitial macrophages could be found in heart autopsy samples, whereas T cells were lower compared to control samples from patients who died from non-infectious causes.^33^

Furthermore, current studies of autopsy samples indicate that the viral mechanism behind the myocardial injury is not an inflammation of the cardiomyocytes themselves but rather of the epicardial endothelium and epicardial nerves, because a lympho-monocytic infliltrate with resulting cardiac neuritis and endothelialitis could be demonstrated.^34,35^

**Figure 2.**
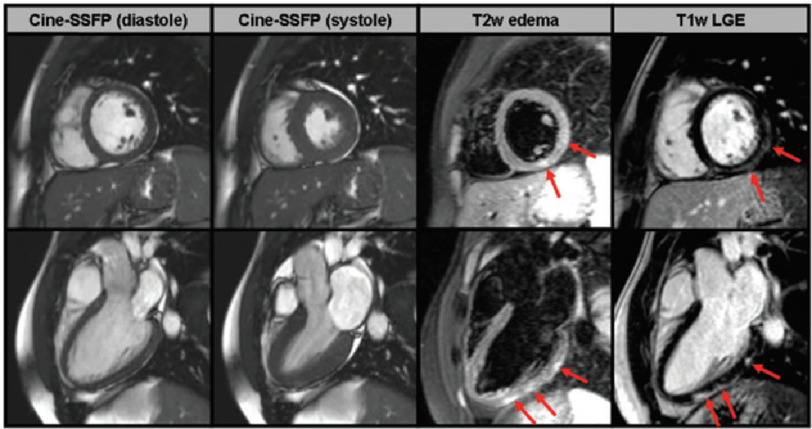
Short-axis (upper line) and long-axis (lower line) CMR images of a young patient with acute myocarditis. In the first two columns, cine-SSFP images are shown in diastole and systole and suggest absence of any wall motion abnormality. In the next column, T2-weighted edema images demonstrate the presence of patchy focal edema in the sub-epicardium of the inferolateral wall (red arrows). In the last column, T1-weighted LGE images demonstrate presence of sub-epicardially distributed LGE (red arrows) which is typical for acute myocarditis^19^.

Even if endomyocardial biopsy is generally still considered to be the gold standard in order to diagnose a myocardial affection, it is rarely performed. Instead, cardiovascular MRI has recently become the preferred non-invasive diagnostic tool of choice as it reveals specific imaging findings for myocarditis (Figure 2)^36^.

### Estimation of AEFI incidence and association with mRNA-vaccination

The estimated incidence of myocarditis recently reported by the Centers for Disease Control and Prevention (CDC) in young males within a few days of the second vaccination was approximately 4.8 cases per 1 million second mRNA-vaccine doses administered^37^. As of 27th September 2021, there were 2,125,702 reports of AEFIs in association with all licensed Covid-19 vaccines in VigiBase. However, because data regarding the total number of administered doses is not collected in VigiBase, and ADR reporting is incomplete, the incidence of mRNA-vaccine induced pericarditis, myocarditis and perimyocarditis for each mRNA-vaccine cannot be calculated. Nonetheless, among 239 different cardiac AEFIs, the term “myocarditis” (which includes the lower-level term “perimyocarditis”) is currently the third most-commonly reported AEFI with 7,520 reports and pericarditis the fourth one with 5,418 reports so far.

In addition, estimating the incidence of myocarditis and its association with COVID-19 mRNA-vaccines is made more difficult because of the application of different diagnostic criteria for pericarditis and myocarditis (for example Brighton Collaboration or European Society of Cardiology). This fact may directly affect the quality of the reported ADRs and reduce their reliability, thereby indirectly affecting the estimated strength of association between mRNA-vaccination and development of pericarditis and myocarditis.

Although there is a risk of developing cardiac ADRs, such as pericarditis or myocarditis after mRNA-vaccination, it is substantially lower than as a result of SARS-CoV-2 infection itself. According to population-based cohort data for the safety of tozinameran, the risk ratio for myocarditis was 3.24 (95% confidence interval [CI], 1.55–12.44) compared to 18.28 (95% CI 3.95–25.12) after SARS-CoV-2 infection^38^. The risk differences were 2.7 events per 100,000 (95% CI 1.0–4.6) vaccinated persons and 11.0 events per 100,000 infected individuals (95% CI 5.6–15.8), respectively^38^. In addition, the course of the disease after mRNA-vaccination seems to be generally milder. Thus, the benefit-risk balance remains undoubtedly positive, as mRNA-vaccines can effectively prevent not only cases of symptomatic illness, hospitalization or death due to SARS-CoV-2, but also these specific cardiac complications^39^.

According to CDC recommendations, patients who develop pericarditis or myocarditis after the first mRNA-vaccination should defer the second dose and, under certain circumstances, consider it again after symptoms, signs, and findings have resolved. It should be noted that, based on current knowledge, a single dose of mRNA-vaccination may not provide sufficient protection against new SARS-COV-2 variants in the general population. Therefore, further efficacy studies comparing the immunization achieved after a single or two mRNA-vaccine doses are still needed^40^.

## Conclusion

In conclusion, we report seventeen clinical cases of myocarditis, perimyocarditis and pericarditis after vaccination with an mRNA-vaccine, which were registered by our pharmacovigilance centre. It is important to continue being vigilant about serious and/or unknown AEFIs and to report them to the national drug authorities. This essential step will allow in-depth assessment of such cases and discrimination between coincidence and causality through confluence of data.
